# Nanoscale observation of PM2.5 incorporated into mammalian cells using scanning electron-assisted dielectric microscope

**DOI:** 10.1038/s41598-020-80546-0

**Published:** 2021-01-08

**Authors:** Tomoko Okada, Tomoaki Iwayama, Shinya Murakami, Masaki Torimura, Toshihiko Ogura

**Affiliations:** 1 Health and Medical Research Institute, National Institute of Advanced Industrial Science and Technology (AIST), Central 6, Higashi 1-1-1, Tsukuba, Ibaraki 305-8566 Japan; 2 Department of Periodontology, Osaka University Graduate School of Dentistry, 1-8 Yamada-oka, Suita, Osaka 565-0851 Japan; 3 Environmental Management Research Institute, National Institute of Advanced Industrial Science and Technology (AIST), 16-1 Onogawa, Tsukuba, Ibaraki 305-8569 Japan

**Keywords:** Imaging, Microscopy, Scanning electron microscopy, Electron microscopy, Nanoparticles, Cellular imaging

## Abstract

PM2.5 has been correlated with risk factors for various diseases and infections. It promotes tissue injury by direct effects of particle components. However, effects of PM2.5 on cells have not been fully investigated. Recently, we developed a novel imaging technology, scanning electron-assisted dielectric-impedance microscopy (SE-ADM), which enables observation of various biological specimens in aqueous solution. In this study, we successfully observed PM2.5 incorporated into living mammalian cells in culture media. Our system directly revealed the process of PM2.5 aggregation in the cells at a nanometre resolution. Further, we found that the PM2.5 aggregates in the intact cells were surrounded by intracellular membrane-like structures of low-density in the SE-ADM images. Moreover, the PM2.5 aggregates were shown by confocal Raman microscopy to be located inside the cells rather than on the cell surface. We expect our method to be applicable to the observation of various nanoparticles inside cells in culture media.

## Introduction

Airborne particulate matter of 2.5 μm or less (PM2.5) is a highly complex and heterogeneous mixtures of various elements, containing chemicals and biologicals coated onto a carbonaceous core^1,2^. PM2.5 has been correlated with various risk factors for acute respiratory infections, chronic respiratory and cardiovascular diseases and type 2 diabetes^3–8^. Typically, air pollution with PM2.5 leads to a high risk of human lung damage^9–14^. In addition, PM2.5 induces systemic effects on several tissues such as the liver and the pancreas^4,15^ and can promote tissue injury via direct effects of particle components leading to oxidative and cellular stress^13,16^.

Mammalian culture cells can be used to analyse biological responses to PM2.5. In previous studies, gene expression analysis was carried out upon direct addition of PM2.5 to cultured cells^2,17–19^. Various changes of protein expression in cultured cells were also caused by exposure to PM2.5^5,17–19^. However, since the effects of PM2.5 on cell functions have been insufficiently investigated, studies are needed on the inner structure of living cells upon addition of PM2.5 to the culture medium at nanometre resolution.

Recently, we have developed a novel imaging technology, scanning electron-assisted dielectric-impedance microscopy (SE-ADM)^20,21^, which enables the observation of various biological specimens in aqueous media without radiation-induced damages at 8 nm spatial resolution^21^. The resolution has been determined as previously described using the edge of protein particles^21^. Analysis at nanometre resolution is very important to study cell functions because the size of protein complexes is of this scale. Biological samples are enclosed in a sample holder composed of two silicon nitride (SiN) films, the upper SiN film being coated with a tungsten (W) layer. When an electron beam (EB) was applied to the W-coated SiN film, the EB was scattered and mostly absorbed by the tungsten layer, so that biological samples were protected from damages by the EB^21^. Our SE-ADM system has been successfully used for high-contrast nano-level imaging of biological specimens such as cultured mouse cancer cells (4T1E/M3)^22,23^ and mouse osteoblastic cells (KUSA-A1)^24^.

In this study, we show that our SE-ADM system can be used to observe PM2.5 incorporated into living mammalian cells in culture media. Our system has allowed direct observation and analysis of the process of PM2.5 aggregation in cells at nanometre resolution. Furthermore, using SE-ADM and Raman spectrum analysis, we have demonstrated that the PM2.5 aggregates are located inside the cells rather than on the cell surface.

## Results

### Observation of OBA9 and 4T1/EM3 cells with PM2.5 under aqueous condition using SE-ADM system

**Figure 1:**
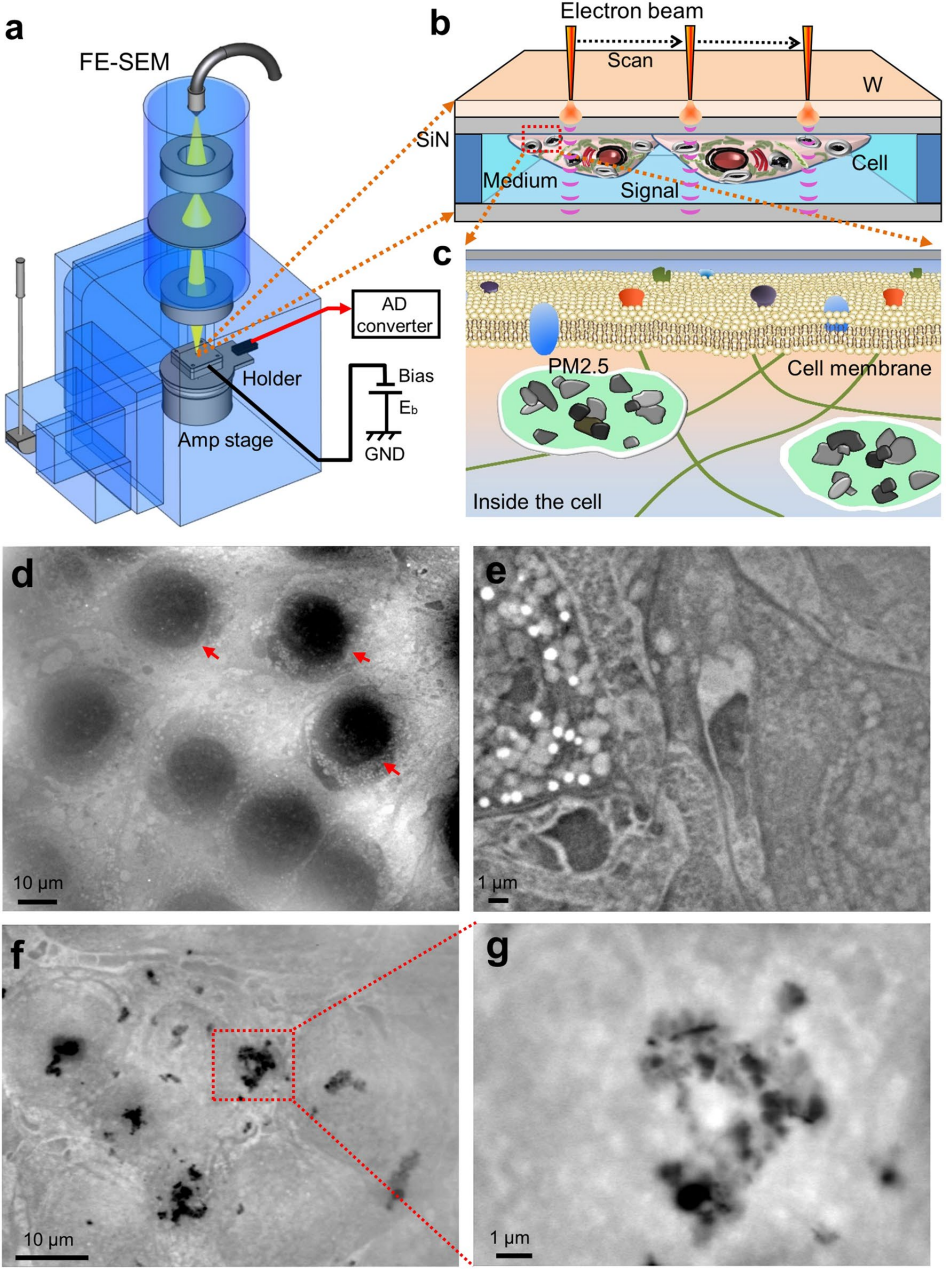
Experimental setup of SE-ADM system and observation of cultured mammalian cells with PM2.5. (**a**) A schematic diagram of the SE-ADM system based on high-resolution FE-SEM. The liquid-sample holder with cultured cells with PM2.5 added was mounted on the pre-amplifier-attached stage, which was introduced into the SEM specimen chamber. The scanning EB was applied to the W-coated SiN film at a low acceleration voltage. The measurement terminal under the holder detected the electrical signal through the liquid specimens. (**b**) Overview of the liquid-sample holder with cultured mammalian cells with PM2.5 aggregates. OBA9 or 4T1E/M3 cells were on the upper SiN film and the W-coated side was irradiated with the scanning EB. (**c**) A conceptual diagram of the PM2.5 aggregates in a cell. (**d**) A low magnification SE-ADM image of untreated OBA9 cells in medium (1000×) with a 7-kV EB and − 9 V bias. Nuclei indicated by red arrows were observed in this image. (**e**) A high magnification image of OBA9 cells (5000×). Intracellular membranes and vesicles were observed. (**f**) An SE-ADM image of OBA9 cells with added PM2.5 at an electron beam acceleration of 7 kV (2000×). Five hours after the addition of PM2.5, the black aggregates were detected in the whole visual field, indicating aggregated PM2.5 in the cells. (**g**) A high-magnification image (10,000×) of a PM2.5 aggregate in the red square in (**f**). Scale bars, 10 μm in (**d**, **f**) and 1 μm in (**e**, **g**).

OBA9, a human gingival epithelial cell line^25,26^, was cultured on a SiN film of 50 nm thickness in a culture dish holder^22^. After the cells formed a confluent monolayer on the SiN film, the holder was separated from the plastic culture dish, sealed in an acrylic holder, and installed in the SE-ADM system (Fig. 1a–c). Cultured OBA9 cells in the holder were kept under atmospheric pressure. While the nucleus was observed in a low-magnification (1000×) image of OBA9 cells (Fig. 1d), intracellular membrane structures and vesicles were discerned at a higher magnification (5000×) (Fig. 1e). Five hours after adding PM2.5 to OBA9 cells in the medium, it was observed that high-density black particles were dispersed in the whole visual field (Fig. 1f). At a high magnification (10,000×) with SE-ADM, PM2.5 aggregates of various sizes were found indeed inside the cells (Fig. 1g, see also Fig. 5). In our SE-ADM system, high density and low-dielectric samples show clear black contrast, as shown in our previous report^27^. PM2.5 is composed of various elements such as carbon and aluminium oxide (see also Fig. 6). It is known that the density of these components is more than 2.0 g/cm^3^, which is much higher than that of cell components, 1.0–1.1 g/cm^3^. Therefore, almost all the PM2.5 particulates and aggregates in the cells or in water show clear black contrast*.*

**Figure 2:**
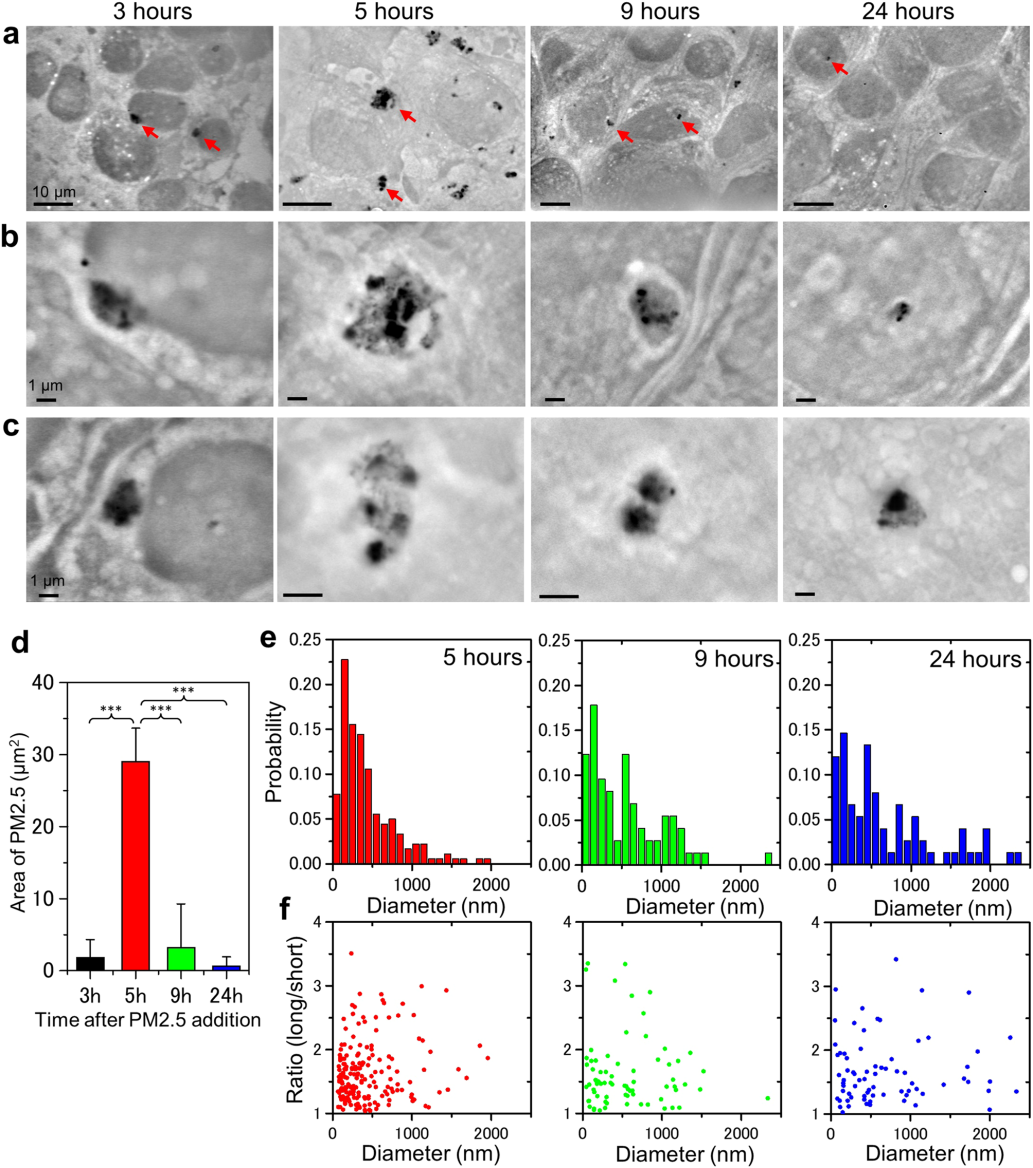
SE-ADM images of OBA9 cells after the addition of PM2.5. (**a**) Low-magnification (1500–2500×) images of OBA9 cells at 3, 5, 9 and 24 h after addition of PM2.5. Black particles are PM2.5 aggregates. (**b**, **c**) High-magnification (**b** 10,000×; **c** 10,000–20,000×) images of the areas indicated by red arrows in (a) indicate the PM2.5 aggregates. PM2.5 aggregates appear to be covered with membrane-like structures at 5 and 9 h after the addition of PM2.5. (**d**) Average area of PM2.5 aggregates in cells at various time. The average area of PM2.5 aggregates in the SE-ADM image (3, 5, 9 or 24 h after addition) was calculated as described in Materials and Methods. p*** < 0.001 (**e**) Distribution of PM2.5 aggregate size in OBA9 cells after 5, 9 and 24 h. After 5 h, the peak of the aggregate diameter histogram was 200 nm and more than 71.4% of the aggregates were smaller than 500 nm. After 9 h, the peak of the aggregate diameter histogram shifted to larger size; 49.3% of the aggregates were larger than 500 nm. (**f**) Point diagrams of the average diameters versus the short and long axis ratio of PM2.5 aggregates in the cells after 5, 9 and 24 h. Scale bars, 10 μm in (**a**) and 1 μm in (**b**, **c**).

Next, we investigated the time course of PM2.5 uptake (Fig. 2). Three hours after addition of PM2.5, a small number of particulates and their aggregates were found inside the cells (Fig. 2a–c, 3 h panels). After 5 h, many PM2.5 particulates were likely to be incorporated into the cells to form aggregates (Fig. 2a–c, 5 h panels). The size and shape of PM2.5 aggregates showed a wide variation in the cells. The area of the aggregates appeared to be larger in the 5 h images compared to the 3 h images (Fig. 2d). The aggregates were observed to be surrounded by a region of low density (Fig. 2b, c, 5 h panels; in the panels, the region looks white). After 9 h, the PM2.5 aggregates were covered with the region of low density, which we assumed to be intracellular membrane-like structures (Fig. 2b, 9 h panels) on the basis of our previous study^24,28^. After 24 h of culture with PM2.5, the number of particulates and aggregates decreased in the whole visual field (Fig. 2a–c, 24 h panels). Some of the PM2.5 particulates once captured by the cells might be exocytosed. The area of PM2.5 in 1000 μm^2^ unit of the image at each time was calculated as seen in Fig. 2d.

Then, we measured the size of PM2.5 aggregates in OBA9 cells using SE-ADM image analysis (Fig. 2e) and a histogram of pixel intensity after contrast inversion was made (Supplementary Figure 1). Five hours after the addition of PM2.5, the histogram of aggregate diameter in OBA9 cells showed a peak at 200 nm and more than 71.4% of the aggregates were smaller than 500 nm (Fig. 2e, left panel). After 9 h, 49.3% of the aggregates were larger than 500 nm in diameter (Fig. 2e, centre panel). After 24 h, 16% of the aggregates were larger than 1400 nm, whereas only a few with such a diameter were detected in 5 and 9 h panels (Fig. 2e right and centre panel). These results suggested that the PM2.5 incorporated in the cells were gradually aggregated and that the size of the aggregates increased. Some aggregates were round while others were elliptical or distorted. Therefore, we calculated the ratio of short axis length to that of long axis of the PM2.5 aggregates in the cells (Fig. 2f). The ratios of all the aggregates after 5 h were found to be between 1 and 3.5 (Fig. 2f left panel). The same tendency was found in the data at 9 and 24 h (Fig. 2f centre and right panels). This result suggested that the aggregate shapes were not affected by the aggregate size. In other words, the shape of PM2.5 aggregates in the cells were not uniform but remained distorted irrespective of elapsed time after the incorporation.

**Figure 3:**
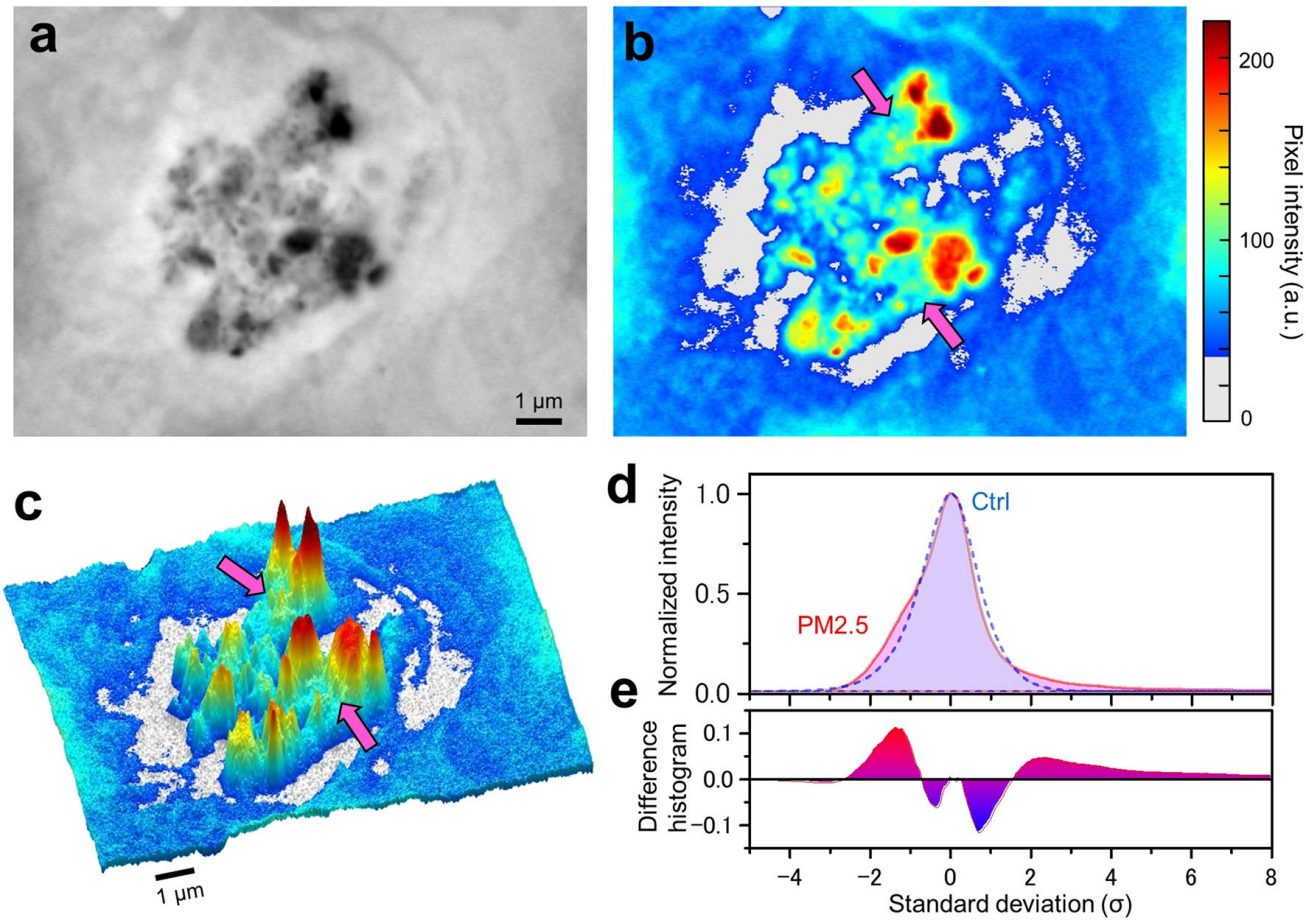
Analysis of pixel intensity at the PM2.5 aggregates in a cell. (**a**) A high-magnification (10,000×) SE-ADM image of a PM2.5 aggregate in OBA9 cells. (**b**) A pseudo-colour map of (**a**) after intensity inversion. Pink arrows indicate the region assumed to be proteins near the PM2.5. (**c**) A 3D intensity-colour map of (**b**). Various shapes of particles were detected in a cell. The PM2.5 aggregates were surrounded by the white region in the image, which was thought to represent intracellular membrane structures. (**d**) Comparison of the distribution of averaged pixel intensity with or without addition of PM2.5 to the cell. The dashed blue curve (control: without PM2.5) displayed a normal probability distribution. The pink curve (with PM2.5) showed positive deviation from the normal probability distribution in areas of higher than 2 standard deviations (σ) and lower than − 1σ. (**e**) A difference histogram between the pink and dashed blue curves. It shows the difference more clearly in the σ areas higher than 2σ and between − 1σ and − 2.5σ. Scale bars, 1 μm in (**a**, **c**).

Our SE-ADM imaging system enables observation of structures of particles and organelles in cells without fixation and staining in aqueous solutions^23^. At a high magnification (10,000×), various shapes of PM2.5 aggregates were found in the cells (Fig. 3a). In SE-ADM images, the PM2.5 aggregates seemed to be surrounded by a white-looking low-density region, which was thought to be intracellular lipid-bilayer membranes as described above. In the previous report^28^, we showed that such a low-density region contained lipids. A pseudo-colour map (Fig. 3b) and 3D map (Fig. 3c) made from Fig. 3a clearly show that the PM2.5 aggregates are surrounded by the low-density white-looking region. Furthermore, the green region indicated by pink arrows is found around the aggregates in red in Fig. 3b. According to our previous results^23,27^ with the SE-ADM system, the protein-rich region appears dark; i.e. pixel intensity is rather high. However, since PM2.5 consists of very high density carbon that does not exist in cells and thus has an extremely high pixel intensity, PM2.5 is observed as truly black particulates (red in the colour image). By contrast, the protein-rich region appears relatively light in the presence of PM2.5 and the colour image should become green. Therefore, it can be inferred that the green region of Fig. 3b is protein-rich.

Figure 3d shows a comparison of the distributions of the average pixel intensities in the image with or without the addition of PM2.5 to OBA9 cells. The average distribution in the case of PM2.5 addition and that of a control were calculated using 11 and 13 SE-ADM images, respectively. Several typical SE-ADM images with and without addition of PM2.5 are shown in Supplementary Figure 2. The average pixel intensity of OBA9 cells without addition of PM2.5 showed a Gaussian distribution (Fig. 3d, dashed blue line). On the other hand, the average pixel intensity after the addition of PM2.5 exhibited an increase at the positions higher than 2σ (Fig. 3d, pink line), which is likely due to PM2.5 particulates. Further, the intensity also exhibited an increase between the -1σ and -2.5σ regions, which is considered to correspond to the lipid-rich membrane of the low-density region shown in Fig. 3b, c. Figure 3e shows the difference histogram between the intensities with and without PM2.5, which is positive in areas higher than 2σ and between − 1σ and − 2.5σ.

**Figure 4:**
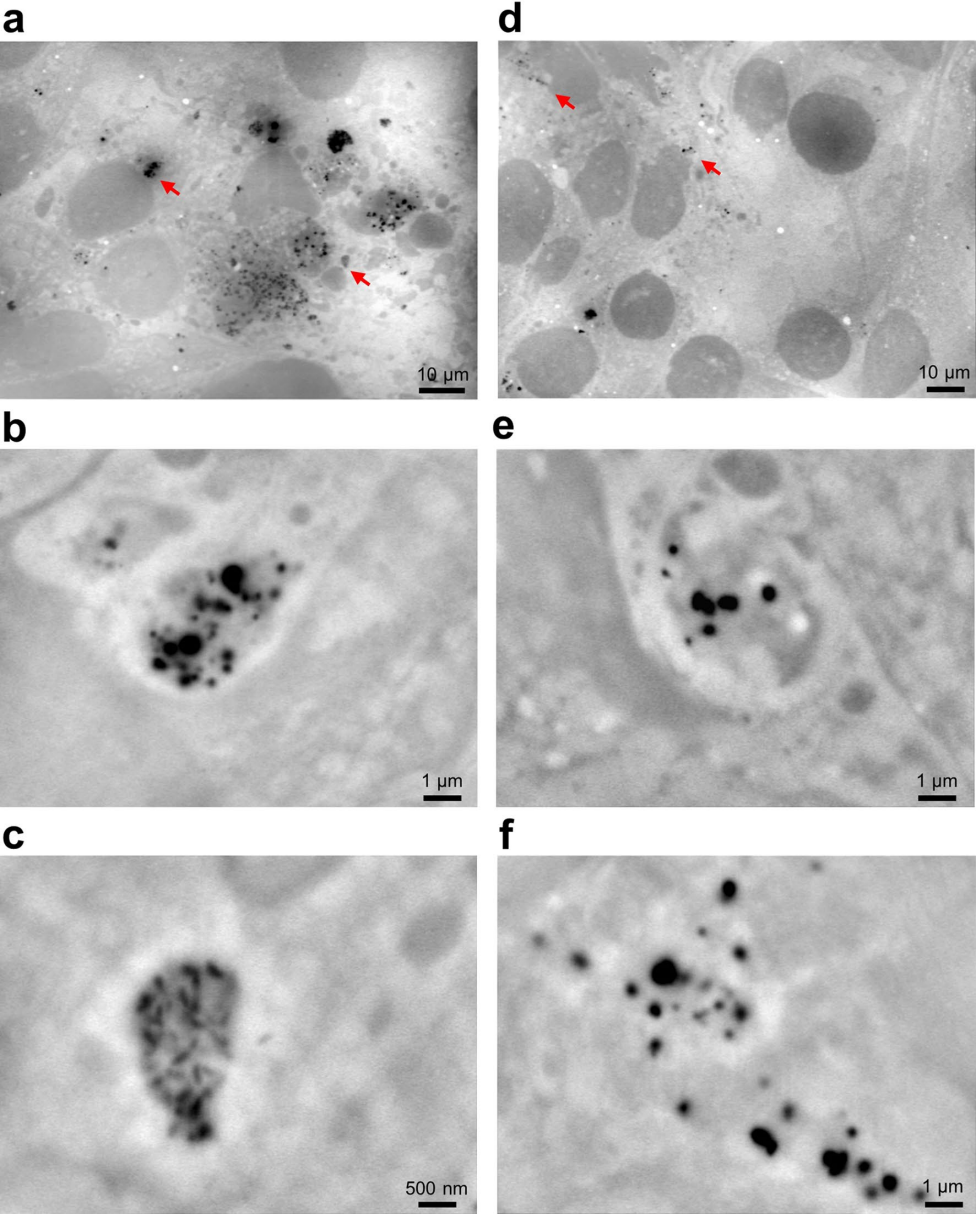
SE-ADM images of 4T1E/M3 cells 3 and 24 h after the addition of PM2.5. (**a**) A low magnification (1200×) image of 4T1E/M3 cells 3 h after the addition of PM2.5. A number of PM2.5 aggregates were seen in the whole visual field. (**b**, **c**) High magnification (**b** 10,000×; **c** 20,000×) images of the areas indicated by red arrows in (**a**). The images show high-density black particulate aggregates covered with light intracellular membrane-like structures. (**d**) A low magnification (1000×) image of 4T1E/M3 cells 24 h after the addition of PM2.5. (**e**, **f**) High magnification (10,000 ×) images of the areas indicated by red arrows in (**d**). Scale bars, 10 μm in (**a**, **d**), 1 μm in (**b**, **e**, **f**), and 500 nm in (**c**).

To further evaluate the PM2.5 incorporation in cells, we added PM2.5 to cultured mouse cancer cells (4T1E/M3) (Fig. 4). Many PM2.5 particulates and aggregates were observed in the whole visual field 3 h after the addition of PM2.5 (Fig. 4a). In high magnification (10,000–20,000×) images, many aggregates were surrounded by the low-density membrane-like region (Fig. 4b, c). The structure of the aggregates was almost the same as that in OBA9 cells (Fig. 2a–c). After 24 h of PM2.5 exposure, the aggregates detected in the cells were fewer than those found in 3 h images (Fig. 4d). In addition, white-looking low-density membrane-like structures appeared to be almost the same as those in the images after 3 h (Fig. 4e, f). These results suggest that the incorporation of PM2.5 may commonly occur in mammalian cells in a quite similar manner in that PM2.5 is surrounded by membrane-like structures.

### Detection of PM2.5 inside OBA9 cells using a confocal Raman microscope

**Figure 5:**
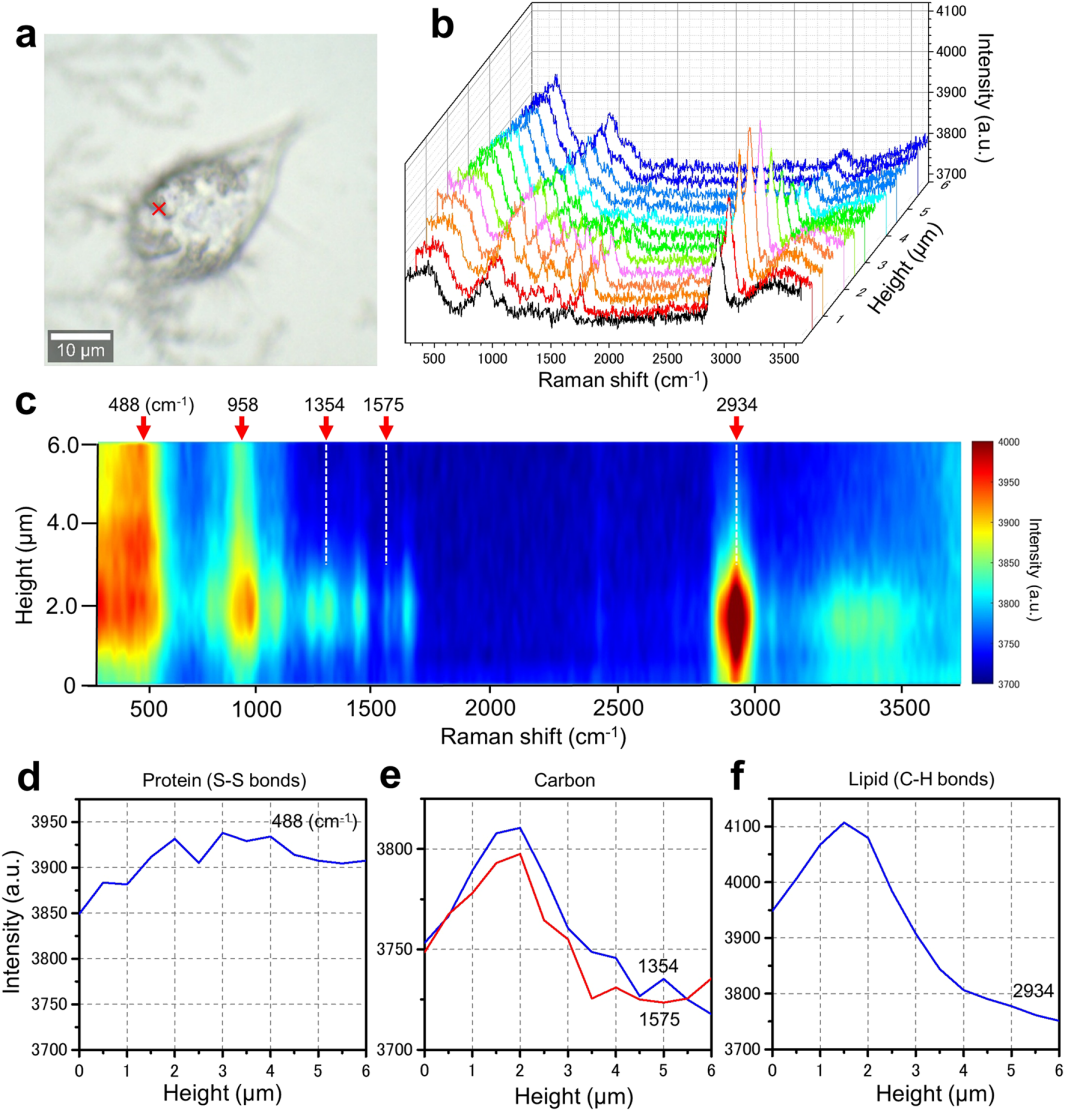
Spatial and spectrum analysis of OBA9 cells added with PM2.5 using confocal Raman spectrum microscope. (**a**) An optical microscopic image of OBA9 cells 5 h after the addition of PM2.5 and fixed with PFA. (**b**) Raman spectra of the position indicated by a red cross in the cell in (**a**) were obtained at 0.5 μm intervals from the glass surface to which the cell adhered (height, distance from the glass surface, 0–6 μm). (**c**) A coloured Raman spectrum map of (**b**). The horizontal and vertical axes are the Raman spectrum intensity and the height from the glass surface, respectively. In this figure, three peaks (488, 958 and 2934 cm^–1^) were discerned. In addition, many small peaks were recognized between 1000 and 1600 cm^–1^. (**d**) A line-plot of the protein peak of S–S bonds at 488 cm^–1^. This signal intensity was rather high throughout. (**e**) Line plots of the carbon peaks at 1354 and 1575 cm^–1^. These signals increased up to 2 μm height and then decreased. (**f**) The line plot of the lipid peak at 2934 cm^–1^. This signal increased up to 1.5 μm height and then decreased. Scale bar, 10 μm in (**a**).

We analysed OBA9 cells treated with PM2.5 using a confocal Raman microscope (Fig. 5). First, we obtained a Raman spectrum of PM2.5 as a powder or a suspension in PBS on a glass slide (Supplementary Figure 3). In either case, the Raman spectrum of PM2.5 showed sharp peaks at 1354 and 1575 cm^−1^, which corresponded to the carbon peaks of charcoal^29^. Therefore, PM2.5 used in the present study is thought to primarily consist of carbon. Next, we investigated the Raman spectrum of OBA9 cells after the addition of PM2.5 (Fig. 5). OBA9 cells were cultured in a glass bottomed dish, treated with PM2.5, cultured for 5 h, and then fixed by paraformaldehyde (PFA) before the measurement. A Raman spectrum at the position of a black particle (indicated by the red cross in Fig. 5a) at the cell centre was obtained with 0.5 μm upward-steps from the surface of the glass bottomed dish (Fig. 5b). The black line in the front in Fig. 5b shows the spectrum at the bottom side of the cell adhered onto the glass.

In the Raman spectra and its colour maps, three peaks at 488, 958 and 2934 cm^−1^ were identified, which corresponded to those of proteins and lipids^30–33^. In addition, a number of small peaks were detected in the area of 1000–1600 cm^−1^ (Fig. 5b, c). As seen in Fig. 5d, the height of the protein S–S bond peak (488 cm^−1^)^30^ gradually increased from the bottom toward the point of 2 μm. The intensity was almost constant from 2 to 6 μm height, which was very likely to be the intracellular region (Fig. 5d) since the thickness of the cell was about 5–10 μm. By contrast, the 1354 and 1575 cm^−1^ peaks due to carbon from PM2.5 steeply increased from the bottom toward the point of 2 μm, and sharply decreased beyond 2 μm (Fig. 5e). These results indicate that the PM2.5 carbon exists inside the cell but not on the cell surface. As for the signal of 2934 cm^−1^ due to lipids, the shape of the signal is almost the same as that of the carbon signal (Fig. 5e, f). We propose that the lipid-rich intracellular membranes surround PM2.5 aggregates. In addition, we obtained the same results at another position of the cell (data not shown). It should be mentioned that the peak of 958 cm^−1^ indicates the collagen backbone CH=CH bending^33^.

### Element analysis of the PM2.5 sample using SEM–EDX system

**Figure 6:**
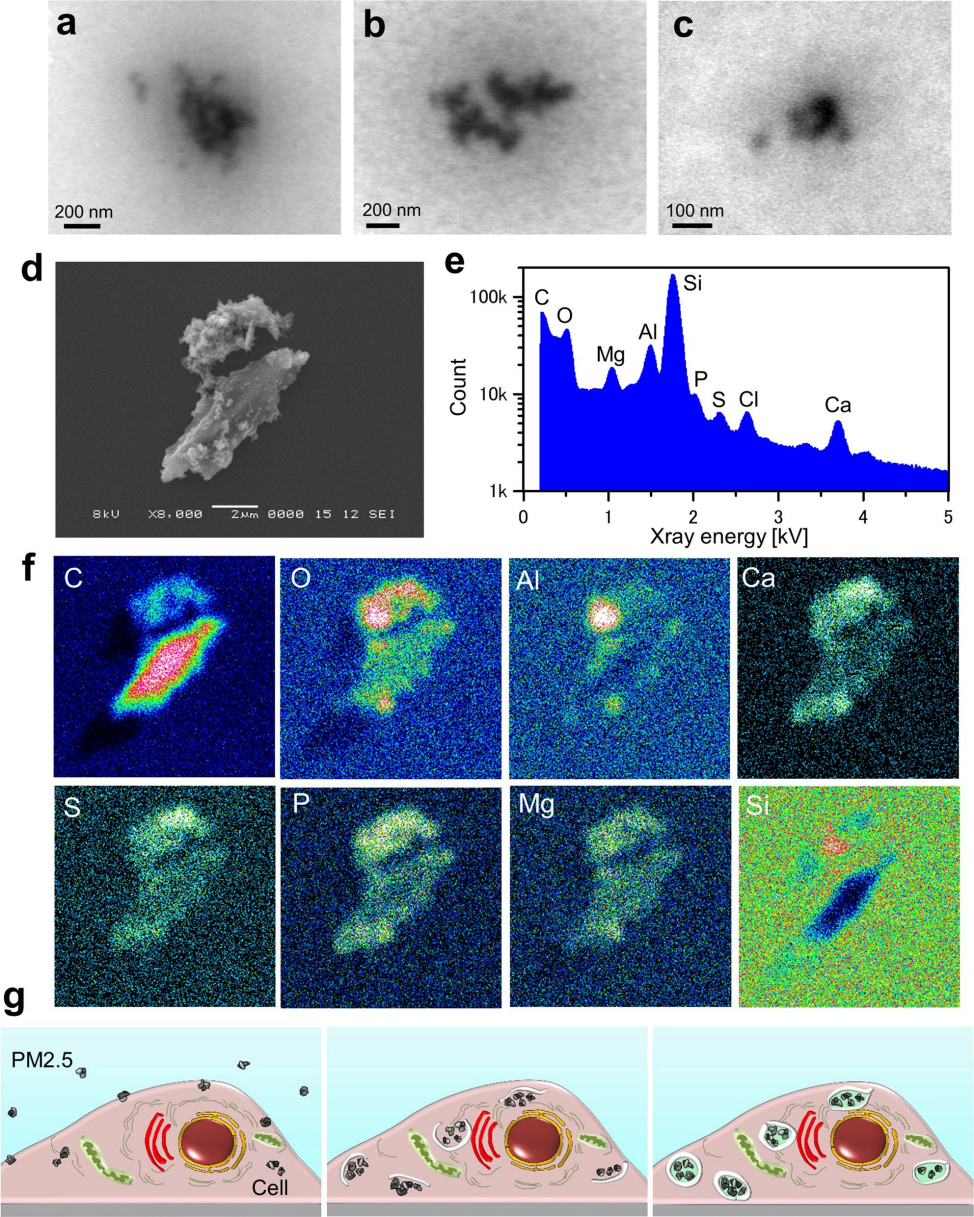
SE-ADM image of PM2.5 suspension and component analysis of PM2.5 using EDX. (**a**,** b**) SE-ADM image (30,000×) of PM2.5 suspension obtained at 3 kV EB. The aggregates were seen to consist of small PM2.5 particulates. (**c**) A high magnification (100,000 ×) image of PM2.5 suspension obtained at 3 kV EB. (**d**) A single dried PM2.5 on a SiN film observed with SEM at 8000× magnification at 8 kV EB. (**e**) A characteristic X-ray spectrum of the PM2.5 in (**d**) using SEM–EDX system. The spectrum shows peaks of carbon, oxygen, magnesium, aluminium, silicon, phosphorus, sulfur, chloride and calcium. (**f**) The element maps of PM2.5 in (**d**). The lower part of the PM2.5 primarily consisted of carbon, while oxygen and aluminium were primary elements in the upper part of the PM2.5. (**g**) Schematic drawing of PM2.5 incorporation into a cell. Shortly after the addition of PM2.5, the particulates are attached to the cell membrane, and a few are taken into the cell (left panel). About 5 h after addition, the incorporated PM2.5 particulates are gradually aggregated and covered with white membrane-like structures (centre panel). At around 9 h, the incorporated particles are covered with the membrane-like structures (right panel). Scale bars, 200 nm in (**a**, **b**), 100 nm in (**c**) and 2 μm in (**d**).

Airborne PM2.5 is a highly complex and heterogeneous mixture of chemical and/or biological components^1^. Here, the composition of the PM2.5 used in this study was analysed by SEM–EDX (Energy Dispersive X-ray Spectroscopy). When the prepared PM2.5 (suspended in PBS, sonicated and filtered through a 5 μm filter) was directly observed by SE-ADM before addition to the cells, particles of various sizes were observed. Various shapes of aggregates with sub-micrometre dimensions were seen, but most of them were smaller than 2.5 μm (Fig. 6a–c). Some other images are shown in supplementary Figure 4a–f. We determined the elements of PM2.5 aggregates on SiN film using a SEM–EDX system (Fig. 6d–f). One aggregate consisted of two big parts associated with a number of tiny particulates of nanometre size (Fig. 6d). An X-ray spectrum in the whole image field showed several peaks, corresponding to carbon (C), oxygen (O), magnesium (Mg), aluminium (Al), silicon (Si), phosphorus (P), sulfur (S), chloride (Cl), and calcium (Ca). The lower part of the aggregate in Fig. 6d was primarily composed of carbon (Fig. 6f, upper panel C). In contrast, the upper part was composed of oxygen, aluminium and calcium, suggesting that the upper part was primarily comprised of aluminium oxide (Fig. 6f). In addition, most of the aggregate surfaces were covered with calcium, sulfur, phosphorus, and magnesium. Since a Si signal was detected due to the presence of the SiN film under the aggregate, the Si signal intensity was low on the aggregate. With a PM2.5 powder sample, we obtained almost the same results as those with the PM2.5 suspended in PBS buffer (Supplementary Figure 5). These results indicated that the prepared PM2.5 sample used in this study was composed of many small particulates consisting of various elements.

## Discussion

Air pollution has been recognized as an important public health problem all over the world^2,3,9^. PM2.5, an air pollutant, is known to be a significant health risk factor^3,9,13^. Mammalian cultured cells can be used for the analysis of biological effects of PM2.5^2,5,18^. In previous studies^11,14,16,18^, it was shown that PM2.5 was incorporated into cells and caused serious defects in the gene expression level and oxidative and cellular stress. Also, changes in cell structures by exposure to PM2.5 were observed using TEM, which can provide detailed analyses of cell structures, showing that the PM2.5 was captured in autophagosomes^14,15,17^. However, the cells in these reports were observed after various preparation procedures, e.g. glutaraldehyde fixation, epoxy resin embedding, thin section slicing and heavy metal staining. Thus, it was desirable to directly observe the effects of PM2.5 in living cells without treatments such as staining and fixation.

Recently, we developed a novel imaging technology, scanning electron-assisted dielectric-impedance microscopy (SE-ADM), which enables direct observation of various intact biological specimens including living cells in aqueous solutions^22–24^.

The density of PM2.5 is much higher than that of the cell components. Therefore, almost all the PM2.5 particulates and aggregates in the cell or in water can be identified in the SE-ADM images. Supplementary Figure 1e is an enlargement of the region between 2σ_L_ to 8σ_L_ of Fig. 3d to show the difference of the normalized intensities in the presence and absence of PM2.5. Supplementary Figure 1f shows the area ratio in the images higher than the threshold of 5σ_L_ with or without PM2.5. When PM2.5 was not added, the statistical probability of particle existence in the medium higher than 5σ_L_ in the images is almost 0% (0.0006). Therefore, we can distinguish the PM2.5 particulates from the cell components by using a threshold of 5σ_L_ in the figures of pixel intensity.

Here we demonstrated incorporation of PM2.5 in human gingival epithelial cells (OBA9) at a nanometre resolution. Five hours after the addition of PM2.5, many high-density particles were detected in the cells (Fig. 1f, g). By the time course analysis (Fig. 2), captured particles in the cells were shown to become aggregates covered with white-looking low-density membrane-like structures (Fig. 2a–c, 5 and 9 h). Shortly after the addition of PM2.5 to the cells, the PM2.5 particulates may be attached to the cell membrane, and only a few particulates were incorporated into the cells (Fig. 6g left panel). After 5 h of the addition of PM2.5 to the cells, the incorporated PM2.5 particulates were gradually aggregated and covered with low-density intracellular membrane-like structures (Fig. 2, 5 hour panel and Fig. 6g center panel). After 9 h, the incorporated PM2.5 particulates were clearly observed to be covered with the membrane-like structures (Fig. 2, 9 h panel and Fig. 6g right panel). Previous studies^17,18^ suggested that PM2.5 induced an autophagy response in cells. Hence, the white-looking membrane-like structures around PM2.5 aggregates in our images might be an autophagosomal structure. Since the spatial resolution of our methods is 8 nm at this time, more detailed structure analysis can be carried out by TEM^17^.

PM2.5 used in this study was an airborne particulate sample (NIES-CRM 28)^34^ that was collected from a central ventilating system of a building in Bejing city centre in China; the period of the collection was between 1996 and 2005. The diameter of the particles was less than 10 μm. In this study, NIES-CRM 28 sample was filtered through a PVDF membrane with pores of 5 μm after being suspended in PBS and being sonicated. The size of the particles was mostly smaller than 2.5 μm (Figs. 2d, 6a–c). It was shown^1,34^ that ordinary PM2.5 samples are composed of various elements such as carbon, sulfur, phosphorus and metals^1,34^. In order to determine the elements of the PM2.5 used here, we employed a SEM–EDX system and found that the PM2.5 was in fact primarily consisted of carbon, sulfur, phosphorus, aluminium, calcium and magnesium (Fig. 6e, f). Carbon of the PM2.5 shows strong Raman peaks at 1354 and 1575 cm^–1^ (Supplementary Figure 3)^29^, different from carbon atoms in cell components such as proteins, fat and DNA^30–33^. Therefore, the carbon peaks above are highly likely due to the PM2.5 inside the cells. In order to unambiguously prove the incorporation of PM2.5 into the cells, the cells were observed at 5 h after the addition by PM2.5 under a confocal-Raman-microscope to detect the carbon peaks (Fig. 5). The carbon peaks at 1354 and 1575 cm^–1^ were in fact detected inside the cells (Fig. 5e), demonstrating that PM2.5 s were indeed incorporated into the cells. Since we fixed and dried the cells before confocal Raman microscopic analysis, we confirmed the same appearance of the cells by low voltage SEM analysis. As shown in supplementary Figure 6, intact cell surfaces could be discerned and very few PM2.5 aggregates were detected on the cell surface after thorough washing when PM2.5 was added to the cells.

In conclusion, we were successful in direct observation of the incorporation of PM2.5 into mammalian cultured cells in aqueous media using SE-ADM system. We also detected the aggregates of PM2.5 in the intact cells. Those aggregates were covered with intracellular membrane-like structures. Also, we demonstrated that the PM2.5 aggregates were not on the cell surface but inside the cells, using confocal Raman microscopy. For providing first insights, the methods described here are very appropriate tools, but for further, more detailed insights, TEM analysis should be performed. Our methods can be applied to observation of various other nanoparticles in cells in aqueous media.

## Materials and methods

### OBA9 and 4T1E/M3 cell culture

OBA9, a human gingival epithelial cell line, was established as previously described^25,26^ and cultured in HuMedia-KG2 (Kurabo Industries Ltd. Osaka, Japan) containing insulin (10 mg/mL), hEGF (0.1 μg/mL), and hydrocortisone (0.67 mg/mL) at 37 °C under 5% CO_2_.

4T1E/M3, a mouse breast cancer cell line, was established as previously described^35–37^ and cultured in high-glucose RPMI-1640 medium (Fuji film Wako, Tokyo, Japan) containing 10% fetal bovine serum (GIBCO Thermo Fisher Scientific) and 20 mM HEPES (Fuji film Wako) at 37 °C under 5% CO_2_. Cells (4 × 10^4^, 1.5 mL/dish) were cultured on a 50 nm thick SiN film in a hand-made culture dish holder^22^.

After 3–4 days of culture, the cells in the holder formed a confluent monolayer on the SiN film in the holder.

### Preparation of PM2.5 and addition to cells

PM2.5 used in this study was Environmental Certified Reference Materials (CRM) No.28, urban aerosols, from National Institute of Environmental Studies (Tsukuba, Japan)^34^. The origin of this material is atmospheric particulate matter collected on filters in a central ventilating system in a building in Beijing city center over a period of 10 years (1996–2005).

The PM2.5 (CRM No.28) was suspended in PBS (20 mg/mL), vortexed for 30 s, sonicated for 4 h (Sonorex TK30, BANDELIN electronic GmbH, Berlin Germany), filtered through Ultrafree-MC (Durapore PVDF 5 μm membrane, Merck Millipore Ltd.) by centrifugation for 4 min at 12,000×*g* at room temperature in a TX-201 centrifuge (Tomy Seiko, Tokyo, Japan). Since an average of 87% (w/w) of PM2.5, the majority of which should be large aggregates, was removed by the filtration, the final concentration of 2.6 mg/mL PM2.5 was used for addition to the cells and in the element analysis by SEM–EDX. After the formation of a cell monolayer, the medium was removed and fresh medium (900 μL/dish) was added to the monolayer. Next, the PM2.5 sample suspension prepared (100 μL/dish) was added to the cell monolayer in the culture dish holder and incubated for 3–24 h at 37 °C under 5% CO_2_. Then the cells in the culture dish holder were washed 3 times with fresh culture medium (1.5 mL each/dish). Next, the Al holder containing cultured cells was separated from the plastic culture dish and attached to another SiN film on a square acrylic plate and sealed to mount on the sample stage of SE-ADM as described previously^22^.

### Liquid sample culture dish holders

The liquid sample holder of the SE-ADM system was made as previously described^21,22^. Briefly, the liquid sample holder comprising of an upper Al holder and lower acrylic resin portion held the cell culture solution at atmospheric pressure between the SiN films^21,22^.

OBA9 cells or 4T1E/M3 cells (1.5 mL/dish) were cultured in the dish holder. These cells formed a sub-confluent or completely confluent monolayer on the SiN membrane in the holder after 3–4 days. Next, the Al holder with a cell monolayer was separated from the plastic culture dish, attached upside down to another SiN film on an acrylic plate and sealed. The Al holder received a voltage bias of approximately − 9 V in the SE-ADM system.

### High-resolution SE-ADM system and FE-SEM setup

The handmade SE-ADM imaging system was attached to a FE-SEM (JSM-7000F, JEOL, Tokyo, Japan, and SU5000, Hitachi High-Tech Corp) (Fig. 1a). The liquid sample holder was mounted onto the SEM stage and the detector terminal was connected to a pre-amplifier under the holder^21^. The electrical signal from the pre-amplifier was fed into the AD converter^21^. The SEM images (1280 × 1020 pixels) were captured at 1000–100,000× magnification with a scanning time of 80 s, a working distance of 7 mm, an EB acceleration voltage of 3–10 kV and a current of 1–10 pA.

SE-ADM signal data from the AD converter were transferred to a personal computer (Intel Core i7, 3.2 GHz, Windows 10) and high-resolution SE-ADM images were processed from the LPF signal and scanning signal using the image-processing toolbox of MATLAB R2018a (Math Works Inc., Natick, MA, USA). The original SE-ADM images were filtered using a 2D Gaussian filter (GF) with a kernel size of 11 × 11 pixels and a radius of 1.2σ. Background subtraction was achieved by subtracting SE-ADM images from the filtered images using a broad GF (400 × 400 pixels, 200σ).

### Raman microscopy

OBA9 cells on the glass bottom dish (Matsunam glass Ltd, Osaka, Japan) were added with PM2.5 and fixed by 4% PFA (Wako, Japan) after removing the culture medium. After washing with water three times, the dried cells were investigated under a confocal Raman microscope using a 532-nm Nd:YAG laser (alpha300R, WITech, Ulm, Germany). Spectra were acquired with a Peltier-cooled charge-coupled device detector (DV401-BV, Andor, UK) with 300 gratings/mm (UHTS 600, WITec, Germany). WITec suite (version 5.0, WITec, Germany) was used for data acquisition. Raman spectral data were plotted using Origin 2015J (OriginLab Co., Northampton, MA USA) and MATLAB R2018a.

### SEM–EDX spectrometric analysis

SEM–EDX spectrometric analysis images of PM2.5 on the SiN film were observed using JSM-5600LV and JED2140 EDX spectrometric systems (JEOL, Japan) at 10 kV and 50–100 pA. The EDX spectroscopic data and the element map images were detected at 10 kV and 200- to 300-pA current of EB. The observation time of spectrum or map was 100 or 1000 s, respectively.

### Calculation of the diameter of PM2.5 aggregates of SE-ADM images

PM2.5 aggregates of SE-ADM images showed extremely high pixel intensity. The diameters of individual PM2.5 aggregates were measured on the assumption that the aggregates could be treated as a circle on a 2D image. In order to determine which were aggregates, regions having a pixel intensity of 5 times standard deviation or more were judged to contain PM2.5 particulates. As seen in the Supplementary Figure 1c, the pixel intensity profile did not show a Gaussian distribution but was skewed to the right side from the median. For this reason, the left side from the median was employed to calculate the standard deviation, σ_L_. Those pixels of the image (Supplementary Figure 1c) with a value of more than 5 times σ_L_ were counted, while the other points were not counted, to convert the image into a binary one (Supplementary Figure 1d) and the diameters of individual PM2.5 aggregate regions was calculated to make Fig. 2e, f.

### Measurement of the PM2.5 region in cells in the time course experiments

The region of individual PM2.5 aggregates in the SE-ADM image (3, 5, 9, 24 h after addition) was calculated using the black region whose pixel intensity was higher than 5 times standard deviation (σ_L_) from the average (Supplementary Figure 1c). For each image, σ_L_ (standard deviation of the left side from the median) was calculated after making the histogram of each image in the same way as shown in Supplementary Figure 1. For generating Fig. 2d, the average areas of PM2.5 aggregates were measured using 6 to 10 images at each time point (6 images for 5 h, 7 images for 9 h, 9 images for 24 h). In each image, the area of PM2.5 aggregates per 1000 μm^2^ image, which should correspond to an approximate area of a single cell (33 μm × 33 μm), was measured. For generating Fig. 2e, f, the numbers of the PM2.5 particles analyzed were 180 for 5 h, 73 for 9 h, and 75 for 24 h, respectively. One-way ANOVA with Origin 2015 J was used for statistical analysis (Fig. 2d and Supplementary Figure 1f).

### Comparison of the SE-ADM image with or without addition of PM2.5 to cells

Using 6–10 SE-ADM images of the cells, typical examples of which are shown in supplementary Figure 2 with (a–c) or without (d–f) addition of PM2.5, pixel intensity profiles were calculated and the averaged peak profile was drawn for the control and that with PM2.5 added. To compare the shape of the peak profiles, the intensity (height) of the peaks was normalized and the standard deviation (σ) from the position of the peak summit was plotted on the horizontal axis shown in Fig. 3d, e.

### Reporting summary

Further information on research design is available in the Nature Research Reporting Summary linked this article.

## Supplementary Information

Supplementary Information.

## Data Availability

All data generated or analysed during this study are presented in this paper or in the Supplementary Information. All the raw data files or spectra are available from the corresponding authors on reasonable request.
