# Supplementary material for: Nanoscale observation of PM2.5 incorporated into mammalian cells using scanning electron-assisted dielectric microscope
Source: Sci Rep. 2021 Jan 8;11:228. doi: 10.1038/s41598-020-80546-0 (PMC7794539; doi:10.1038/s41598-020-80546-0)
Supplement: Supplementary file 1 — Supplementary Information. [file 41598_2020_80546_MOESM1_ESM.pdf]

Supplementary information

Nanoscale observation of PM2.5 incorporated into mammalian cells using scanning electron-assisted dielectric microscope

Tomoko Okada[1], Tomoaki Iwayama[2], Shinya Murakami[2], Masaki Torimura[3], Toshihiko Ogura[1]*

[1]Health and Medical Research Institute, National Institute of Advanced Industrial Science and Technology (AIST), Central 6, Highashi, Tsukuba, Ibaraki 305-8566, Japan

[2]Department of Periodontology, Osaka University Graduate School of Dentistry, 1-8 Yamada-oka, Suita, Osaka 565-0851, Japan

[3]Environmental Management Research Institute, National Institute of Advanced Industrial Science and Technology (AIST), 16-1 Onogawa, Tsukuba, Ibaraki 305-8569, Japan

*Corresponding author: Toshihiko Ogura
Health and Medical Research Institute, National Institute of Advanced Industrial Science and Technology (AIST),
Higashi 1-1-1, Tsukuba, Ibaraki 305-8566, Japan
Tel.: +81-29-861-3408, Fax: +81-29-861-2677
E-mail: t-ogura@aist.go.jp

This PDF file includes:

Supplementary Figs 1 to 6

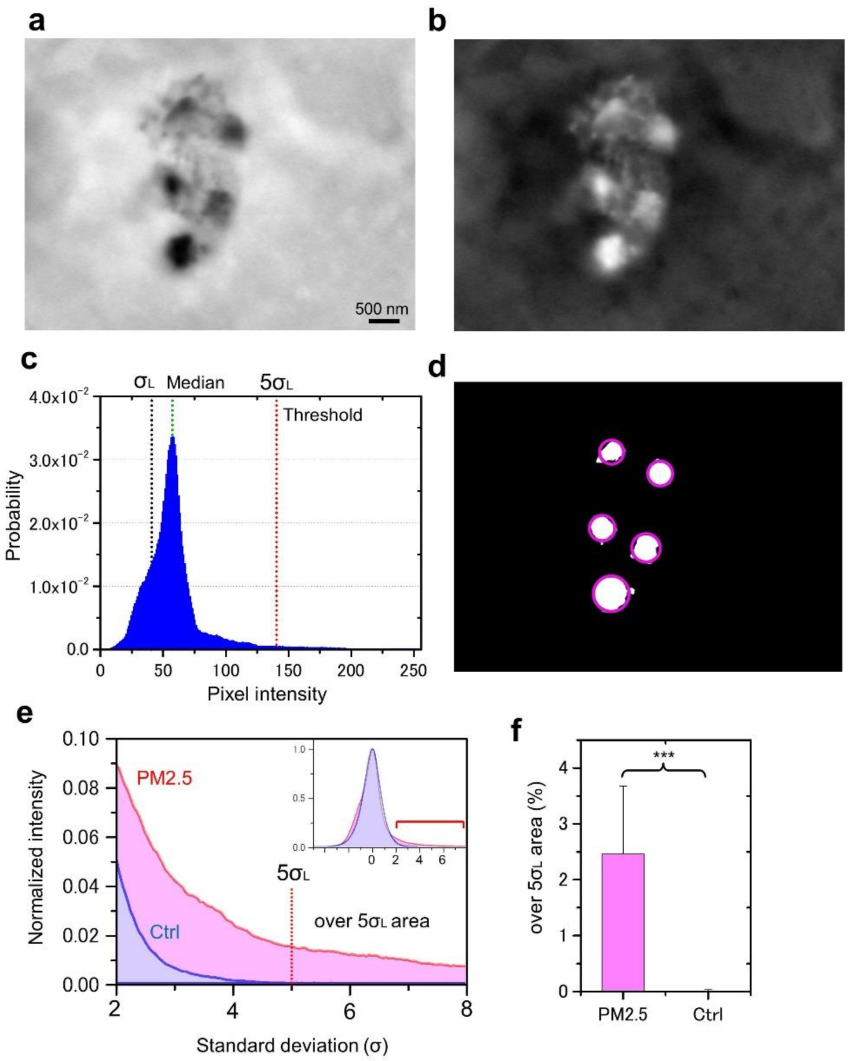

Supplementary Figure 1. Calculation of the diameter and normalized intensity of PM2.5 aggregates in cells using SE-ADM image. (**a**) An SE-ADM image of PM2.5 in an OBA9 cell at 10,000× magnification and 7 kV EB. Scale bar, 500 nm. (**b**) A contrast inversion image of (a). The white particulates are those of PM2.5 in an OBA9 cell. (**c**) A histogram of the pixel intensity of (b). The standard deviation of pixel intensity (σ_L) was calculated using the side with a lower intensity than the median value (distribution peak). (**d**) The PM2.5 aggregate areas were detected by a binary image with intensities higher than 5 times σ_L. The white regions of the binary image indicate PM2.5 aggregates. The magenta circles show approximate contours of the PM2.5 aggregate area calculated from the sum of pixels in the white regions. (**e**) Enlargement of the area between $2\sigma_L$ to $8\sigma_L$ of Fig. 3d inserted in (e). With the addition of PM2.5, the pixel intensity was significantly higher than that of control. (**f**) The percentage of the intensity over $5\sigma_L$ area in (e) was calculated. With PM2.5, the intensity was incomparably higher than that of control. $p^{***}<0.001$.

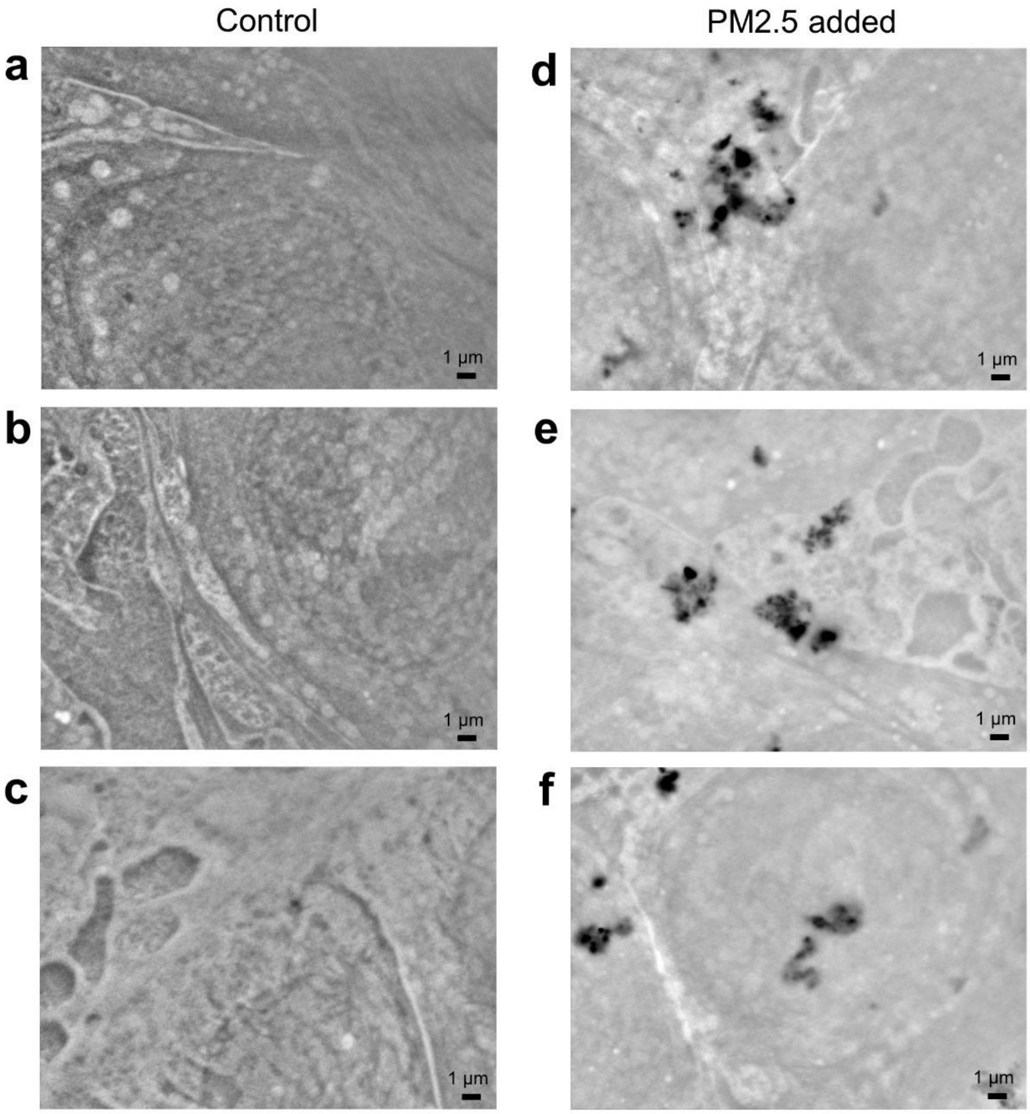

Supplementary Figure 2. Typical SE-ADM images of OBA9 cells with or without PM2.5. (**a–c**) Images of OBA9 cells without PM2.5. These images show complex structures of intracellular membranes and vesicles in OBA9 cells. Note that there are no black particulates. (**d–f**) SE-ADM images of OBA9 cells 5 hours after addition of PM2.5. These images show black particulates due to PM2.5 aggregates. Scale bars, 1 μm in (a–f).

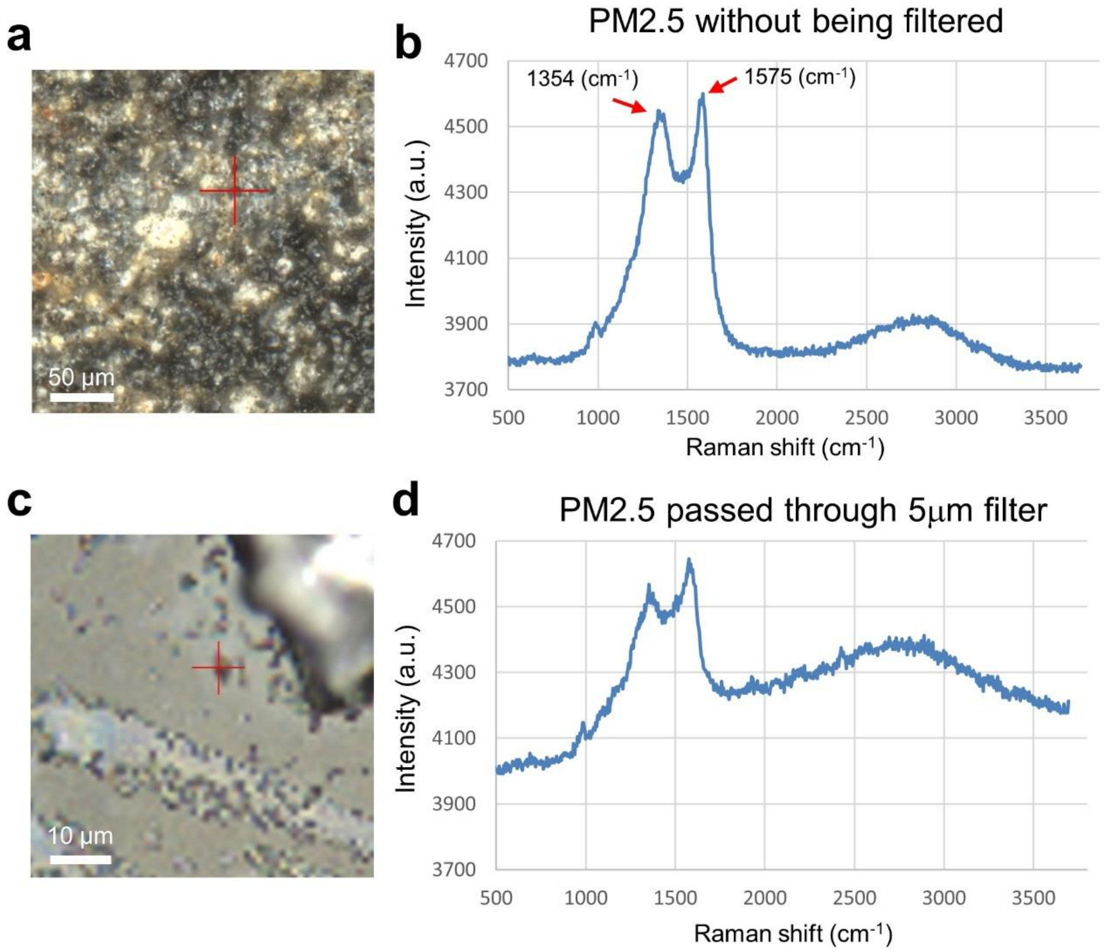

Supplementary Figure 3. Raman spectrum of PM2.5 powder and PM2.5 suspended in a solution using confocal Raman microscopy. (**a**) An image of a PM2.5 powder sample obtained using the optical microscope mode of the Raman system (400× magnification). (**b**) Raman spectrum of the black particulate at the red cross indicated in (a). Raman peaks of 1354 and 1575 cm^{-1} are carbon signals. (**c**) An image of a suspended PM2.5 sample obtained using the optical microscope mode of the Raman system (400× magnification). (**d**) Raman spectrum of the black particulate at the red cross indicated in (c). This spectrum indicates the presence of carbon. Scale bars, 50 μm in (a) and 10 μm in (c).

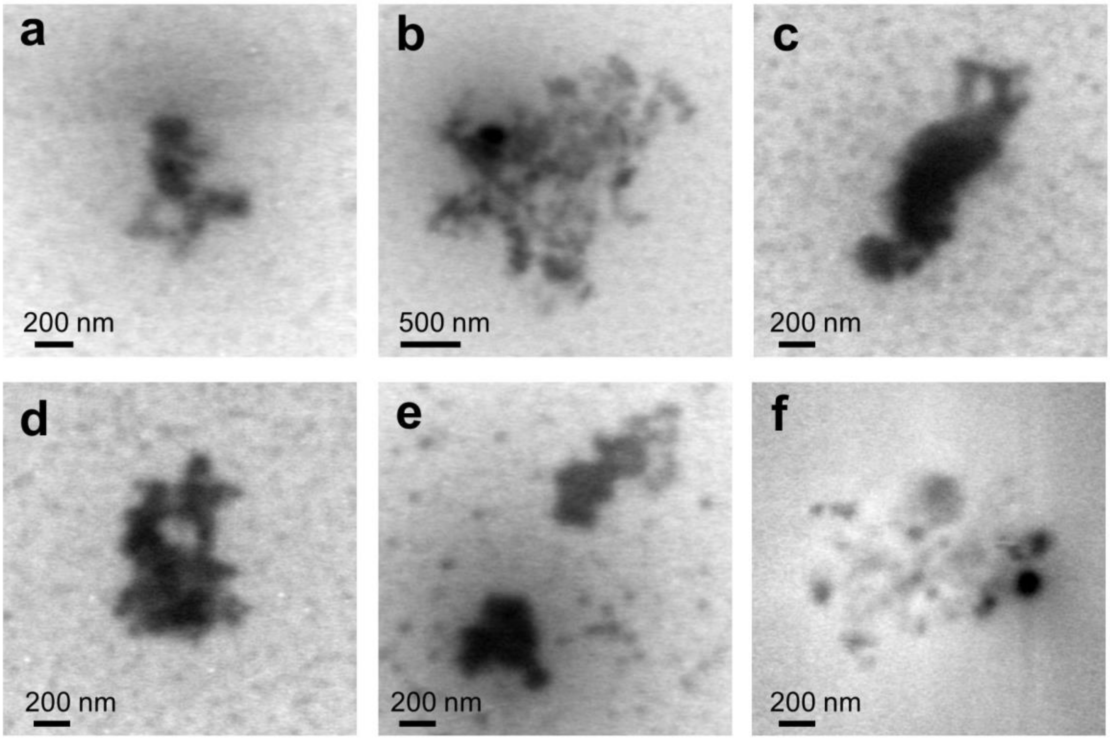

Supplementary Figure 4. SE-ADM images (20,000–30,000×) of PM2.5 suspension in PBS obtained at 3 kV EB. The aggregates were seen to consist of small PM2.5 particulates. Scale bars, 200 nm in (a, c–f) and 500 nm in (b).

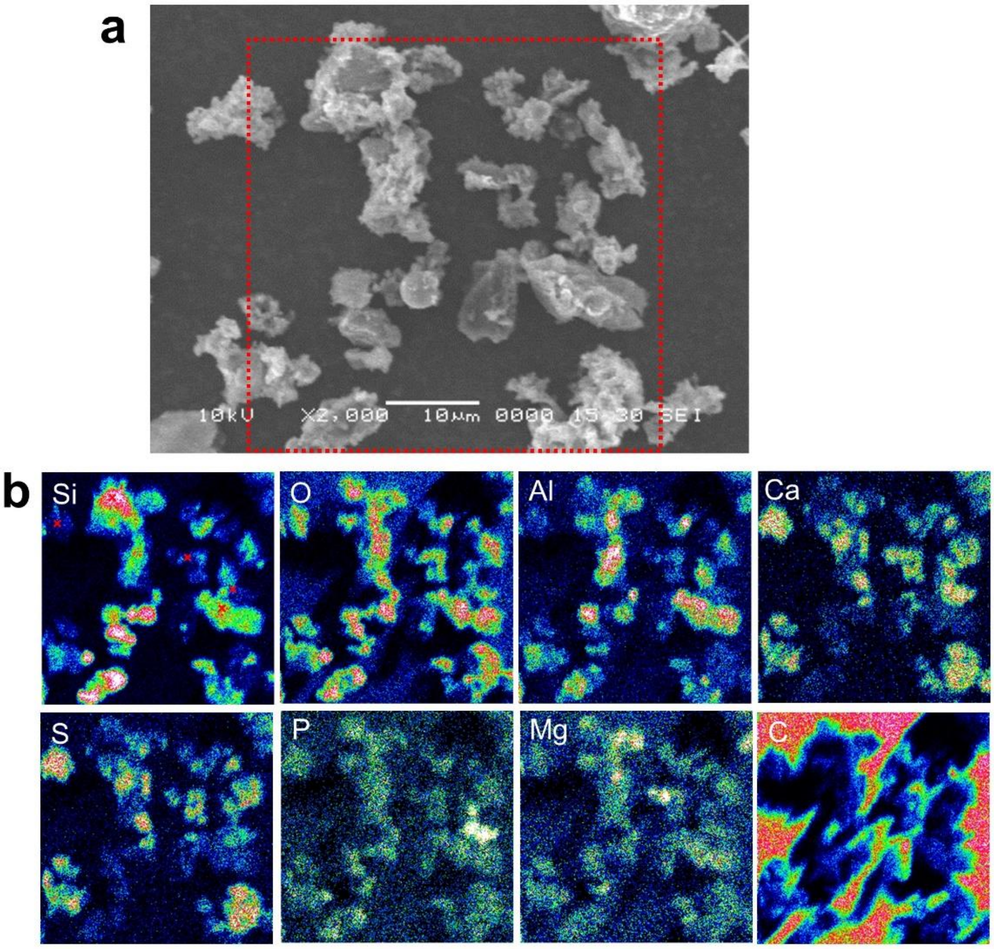

Supplementary Figure 5. Component analysis of PM2.5 particles on a carbon tape by EDX. (**a**) An image of PM2.5 powder on a carbon tape using SEM at 2,000× magnification and 10 kV EB. Scale bar, 10 µm. (**b**) Element maps of PM2.5 powder in (a). PM2.5 primarily consists of 8 elements, i.e. Si, O, Al, Ca, S, P, Mg and C.

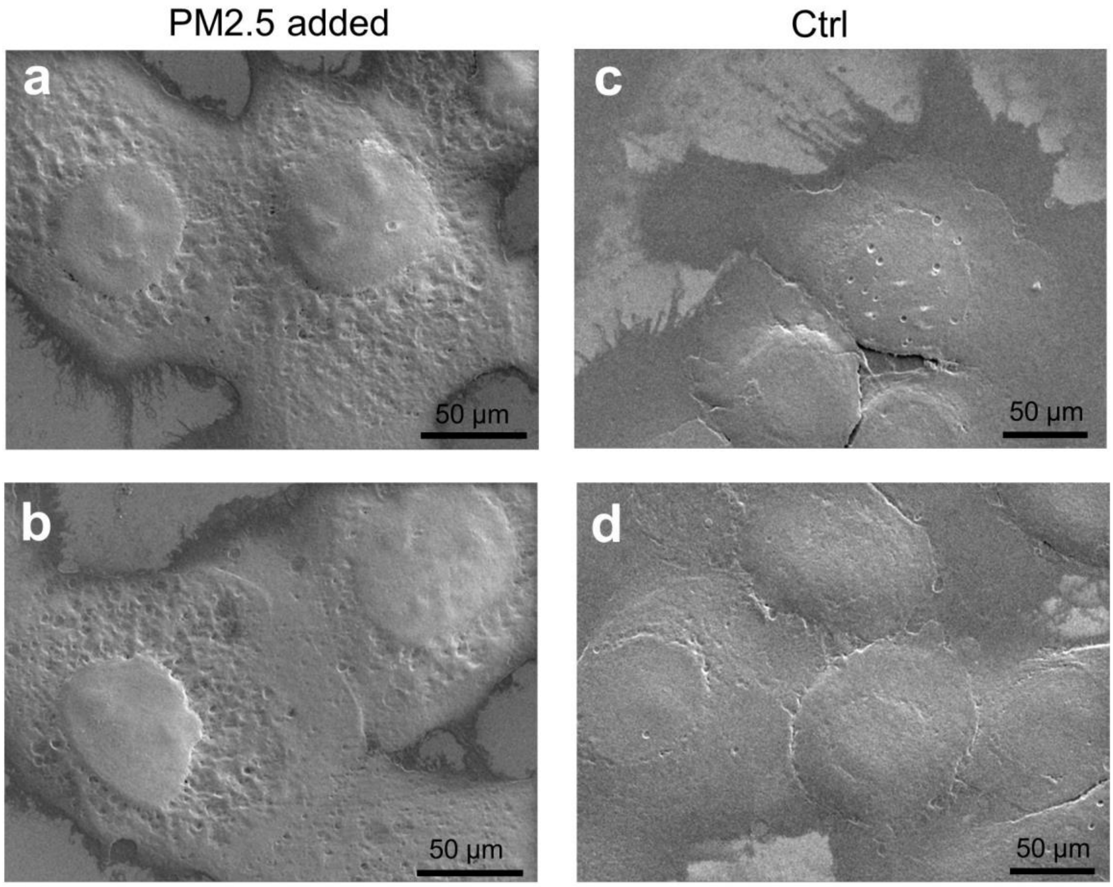

Supplementary Figure 6. SEM images of OBA9 cells obtained at 1 kV EB (2,000–2,500×) by the same protocol of Figure 5 to confirm the intactness of the cell surface. Scale bars, 50 μm in (a–d).
